# Development of a smoking simulation machine to evaluate the effects of smoking on the color change of dental restorative materials

**DOI:** 10.1038/s41598-025-96898-4

**Published:** 2025-04-28

**Authors:** Ali Nadm Hmood, Maha Mohamed Ahmed Ebaya, Abeer El-Sayed El-Embaby

**Affiliations:** Department of Conservative Dentistry, Faculty of Dentistry, Mansoura University, Mansoura, Egypt

**Keywords:** Smoking simulation, Dental materials, Resin-based composites, In vitro testing, Color change, Tobacco effects, Dentistry, Engineering

## Abstract

To develop a smoking simulation machine, the study evaluated the effects of conventional and electronic cigarette smoke on the color stability of resin-based composites. Two types of nanohybrid resin-based composites were divided into two groups based on the material and subgroups according to the kind of exposure: electronic cigarettes, conventional cigarettes, and control. The exposure was by using a newly developed smoking simulation machine, and color change was the primary outcome, measured with a spectrophotometer and calculated using ΔEab. The results showed significant differences in color change were observed between the groups and subgroups (*p* < 0.001). Specimens exposed to conventional cigarettes exhibited more significant discoloration compared to those exposed to electronic cigarettes and control. The custom-made machine demonstrated the ability to simulate smoking conditions and their effects on dental materials. The machine provides a standard and controlled method for evaluating smoke’s effects on restorative materials, while not all materials exhibited similar reactions under the same smoking conditions. The machine impacts dental materials testing and accurately simulates the oral environment, providing insights into material performance that helps formulate materials that resist tobacco smoke, improve restoration durability with esthetics, enhance patient outcomes, and guide material selection.

## Introduction

Examining dental materials and assessing the behavior of these materials is essential in studying their properties and improving them. Although in vivo tests are critical to knowing their real-time physical properties, in vitro tests are still crucial in studying them in a controlled environment^1^. One of the most tested materials in dental schools was resin-based composites (RBCs) and their long-term stability in oral conditions^2^. RBCs are popular for dental restorations because they match natural tooth color and offer several esthetic benefits^3^. Despite the well-known health concerns associated with smoking, there are about 1.3 billion people worldwide who use tobacco products, according to the World Health Organization. Studying the effects of smoking on the esthetic and physical properties of dental materials is an ongoing endeavor. One of the disadvantages of these materials is their reduced durability and esthetic performance when exposed to smoke. They lose some of their clinical efficacy and the pleasure of their patients when they become discolored, rough, and possibly mechanically deteriorated due to smoking^4,5^.

Prior studies on the impact of smoking on dental materials have identified critical problems, including discoloration and surface roughness^4,6–18^. While existing in vitro models of smoke exposure machines have provided valuable data, they fail to accurately represent the actual situation due to the limitations of the machines used. Most of the existing custom-made devices^6–11,13,14,16–18^, lack essential features, such as temperature-controlled artificial saliva and precise aerodynamics, which are necessary to ensure that all the specimens receive a consistent amount of smoke exposure^19^. Although commercial devices like the Vitrocell System (GmbH, Waldkirch, Germany), designed for biological studies^20^, may provide some reliability^4,12,15^, they do not adequately simulate oral environmental consistency^19^, and are unable to provide uniform smoke exposure. In addition, these devices cannot be adapted for dental material testing, particularly in assessing long-term effects such as color change and other properties^4,6,19^. As a result of these limitations, they cannot be used to evaluate the durability of materials over time precisely.

This paper aims to solve the limitations of existing models, such as considering the smoking cycles, the aerodynamics of the smoke/air, and temperature-regulated artificial saliva by introducing a novel smoking simulation machine. This innovative device allows for precise and repeatable in vitro testing by connecting laboratory simulations with real-world conditions. Dental restorative materials, especially RBCs, can be subjected to a series of tests to determine how different smoking sources affect their durability and esthetic stability. These improvements might lessen the variance between in vitro and in vivo studies so that this device can provide a solid foundation for such investigations and research.

## Materials and methods

### Materials

The study utilized two types of RBC materials and various products to simulate exposure conditions and other relevant materials for testing, as listed in Table 1.

**Table 1:** Materials used in this study.

Material	Manufacturer	Classification	Composition	Batch Number
Neo Spectra ST-HV	Dentsply, Konstanz, Germany	Nanohybrid (multi-shade) (A2 shade)	Matrix (Urethane-modified Bis-GMA, TEGDMA), filler (78–80 wt% pre-polymerized, barium glass, and YbF3, size 0.1 to 3.0 µm), photoinitiator system (CQ, BPI, EDMAB)	2311000229
Charisma Diamond One	Kulzer GmbH, Hanau, Germany	Nanohybrid (single-shade)	Matrix (TCD-DI-HEA, UDMA, TEGDMA), filler (81 wt% B2O3-F-Al2O3-SiO2, size 5 nm-20 µm), photoinitiator system (CQ and EDMAB)	M010025
Juul (electronic cigarette)	Juul Labs, San Francisco, USA	4th generation of electronic cigarette	Juul device rechargeable Juul pods are prefilled disposal cartridges (cartridges: 5% nicotine, Virginia tobacco flavor) contained: nicotine 55.0 (mg/ml), PG 365.0 (mg/ml), VG 710.5 (mg/ml), PG/VG ratio 32/63	ME27SA06A
Marlboro Red (conventional cigarette)	Philip Morris, Neuchâtel, Switzerland	Conventional cigarette	Tar 10 mg, nicotine 0.8 mg, carbon monoxide 10 mg, tobacco, water, sugars, PG, glycerol, licorice extract, diammonium phosphate, ammonium hydroxide, cocoa, cocoa products, carob bean and extract, natural and artificial flavors	CN34233002

Bis-GMA: Bisphenol A-glycidyl methacrylate; UDMA: Urethane dimethacrylate; TEGDMA: Triethylene glycol dimethacrylate; TCD-DI-HEA: Bis-(acryloyloxymethyl) tricyclo [5.2.1.02,6] decane; SiO2: Silicon oxide (silica); YbF3: Ytterbium trifluoride; B2O3-F-Al2O3-SiO2: Boro-fluoro-aluminosilicate; CQ: Camphorquinone; EDMAB: Ethyl-4-(dimethylamino) benzoate; BPI: Bis(4-methyl-phenyl)iodonium hexafluorophosphate; µm: Micrometer; nm: Nanometer; PG: Propylene glycol; VG: Vegetable glycerin; Tar: Total aerosol residue.

### Methods

The research protocol for this current study was formally approved by the Ethical Institution Committee, Faculty of Dentistry, Mansoura University, Egypt, under A0401024CD.

### Sample size calculation

The sample size determined was predicated on the differential effects of cigarette smoking on the color change of dental RBCs, as derived from prior research^15^. Utilizing G Power software version 3.1.9.7 to determine sample size based on an effect size of 2.23, employing a two-tailed test with an alpha error of 0.05 and a power of 90.0%, the total determined sample size will be 6 per subgroup.

### Group allocation and randomization

Thirty-six (*n* = 36) specimens were allocated into two main groups (*n* = 18) according to the restorative material used. Group NS (Neo Spectra ST-HV, Dentsply) and group CD (Charisma Diamond One, Kulzer). Each main group was randomly allocated by using serially sealed, numbered, opaque envelopes^21^. Consequently, they were assigned into three subgroups (*n* = 6) according to the type of exposure agent: Subgroups I (NSI and CDI) were exposed to electronic cigarettes (e-cigarettes, Juul), subgroups II (NSII and CDII) were exposed to conventional cigarettes (c-cigarettes, Marlboro Red), and subgroups III (NSIII and CDIII), which served as the control group, had no exposure that incubated with artificial saliva for the exposure period.

### Specimen preparation

A Teflon mold was used to create standard-size discs with a 2mm thickness and 10mm diameter. A transparent celluloid strip was placed on a glass slab, and the mold was filled with restorative material using the bulk-pack technique. RBCs were adapted using a modeling instrument (CompoRoller, Kerr, Switzerland) to ensure contact with the mold and air entrapment. The mold was covered with another strip and glass slab to avoid contamination. A calibration weight of 500 g was placed on the glass slab for 20 s to standardize pressure and uniform stress distribution^22^. Excess restorative material was removed, and another strip was applied to create a smooth outer layer and air inhibition layer^23^. The mold was exposed to a light-curing device for 20 s, using a light-emitting diode (LED) with an intensity of 1500 mW/cm^2^ (Radii Xpert, SDI Limited, Bayswater Victoria, Australia).

After polymerization, specimens were stored in a dark container with distilled water in an incubator (DS20, BioStep, Egypt) at 37 ± 1 °C for 24 h^24^. After that, the specimens underwent a delayed finishing and polishing process^25^. This was achieved by Enhance Finishing System and Enhance PoGo Polishing System (Dentsply/Caulk, Milford, DE, USA), with the low-speed handpiece (NSK, Tokyo, Japan), linked to a micro-motor (Strong 90-108E, Saeshin, Daegu, Korea), at 20,000 rotations per minute for 2 s, with both finishing and polishing procedures for each specimen without water spray^26^. To decrease the surface roughness as much as possible and to remove the resin-rich layer^27,28^.

### Custom-made smoking simulation machine for in vitro testing of dental restorative materials

**Fig. 1:**
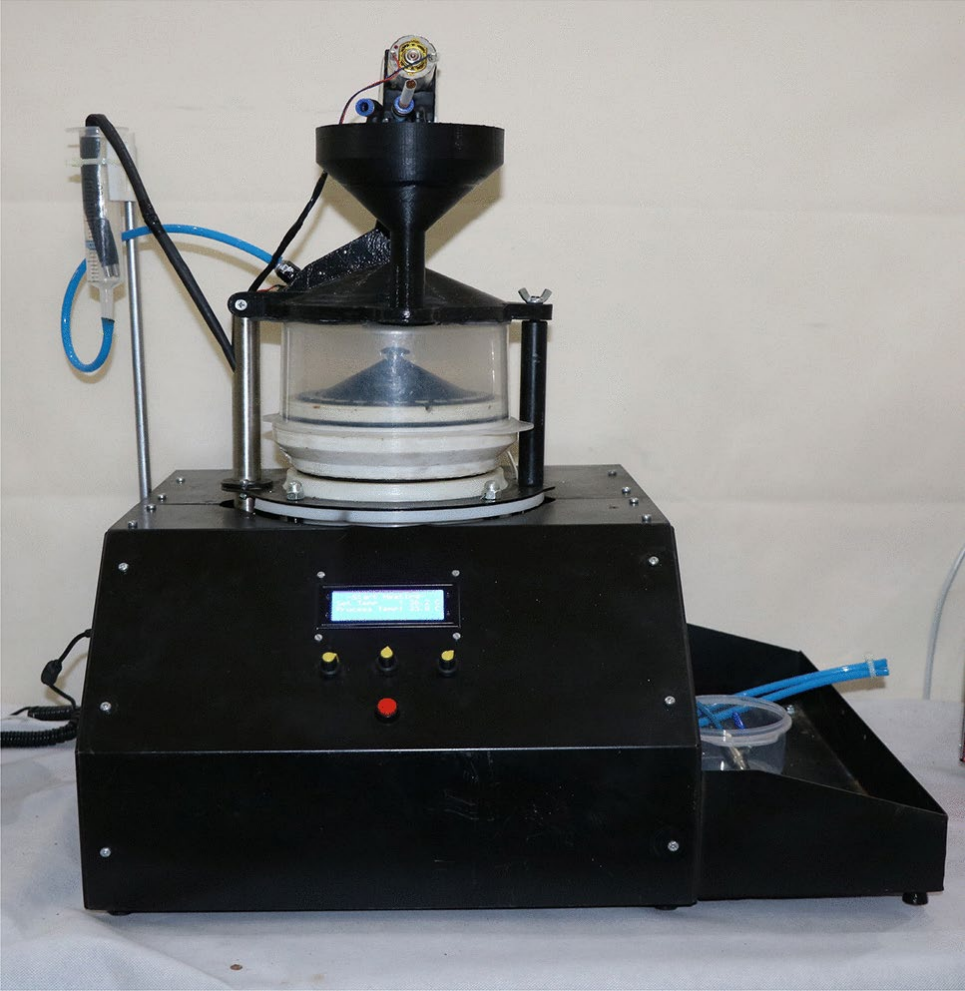
Custom-made smoking simulation machine for in vitro testing of dental restorative materials. (This figure shows the custom-made smoking simulation machine designed for the study).

**Fig. 2:**
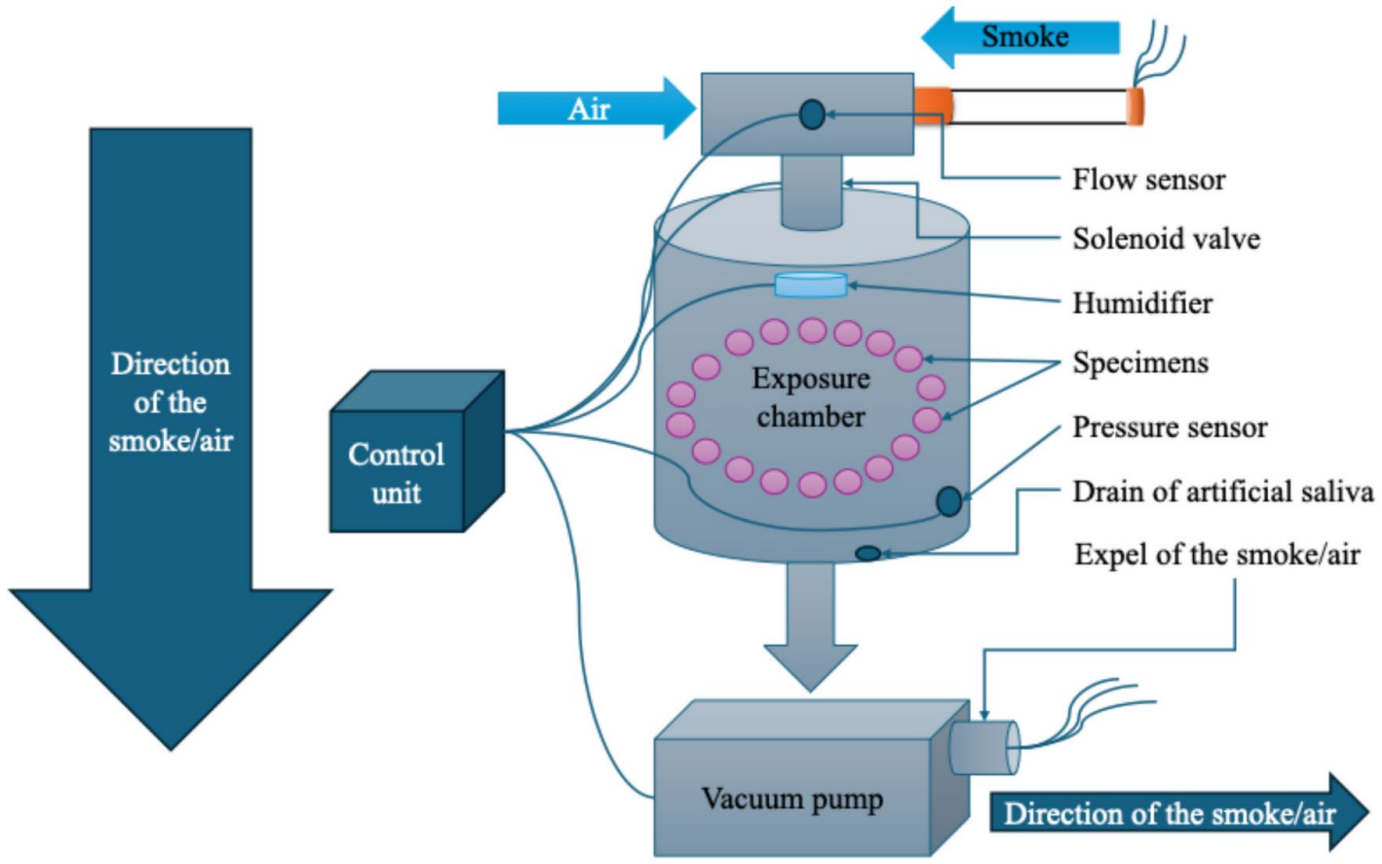
Diagram showing the system components and workflow. (This diagram illustrates the different components of the smoking simulation machine and their interconnected workflow. It highlights the air/smoke inlet system, solenoid valve, exposure chamber, and the sensors used for monitoring pressure, flow, and temperature. The diagram provides a clear overview of how the system operates to simulate smoking cycles).

**Fig. 3:**
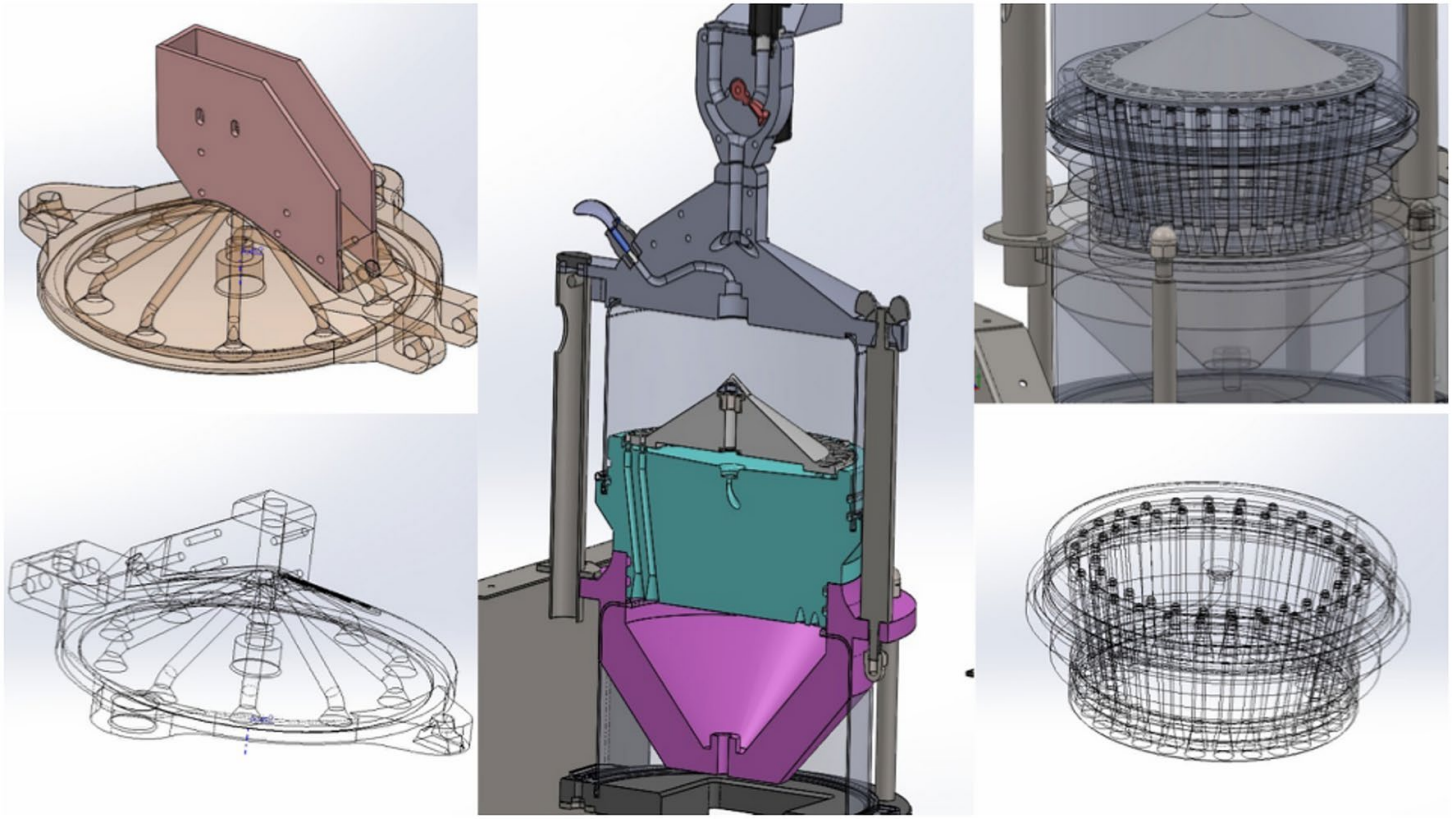
Connections within the machine created by SolidWorks software. (This figure created by SolidWorks software version 2022 https://www.solidworks.com, focuses on the internal connections of the machine, specifically the tubing and connections between the components. It shows how smoke or air is directed through the system and into the exposure chamber. The flow of smoke and the connections between various components are crucial for maintaining consistent exposure to the dental materials).

**Fig. 4:**
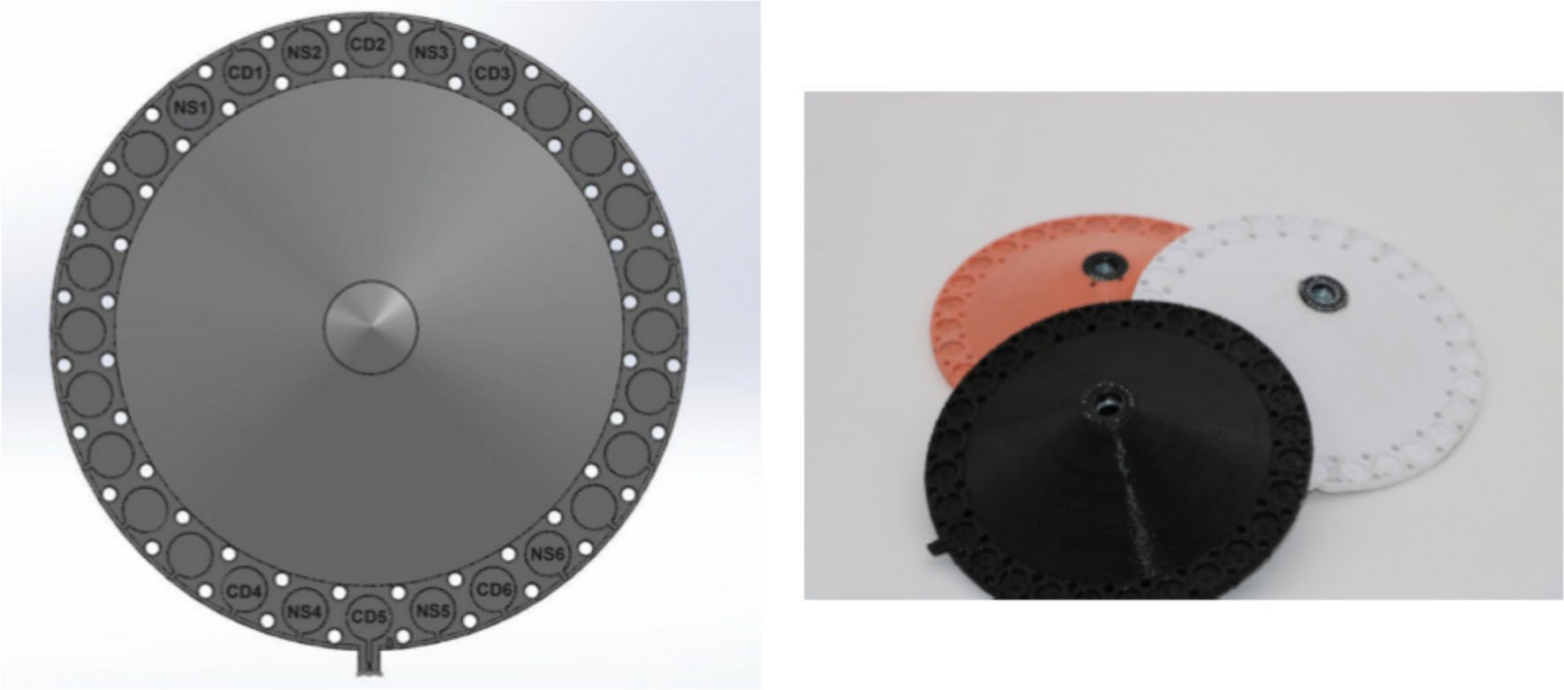
Specimens holders (I: white, II: black, III: pink). (This figure presents the specimen holders used in the experiment. These holders are designed to hold the dental specimens securely within the exposure chamber. Each holder is color-coded (white, black, and pink) to differentiate between the three subgroups (e-cigarette, conventional cigarette, and control). The holders ensure uniform exposure to the smoke and maintain the proper positioning of the specimens).

The smoking simulator machine (Fig. 1) was specially designed and built to closely replicate the smoking conditions for testing dental materials. The machine consists of several key components (Fig. 2), which interact to create a controlled system. This machine consists of:

- Inlet for the air or smoke with cigarette holder that allows either horizontal (c-cigarette) or vertical placement (e-cigarette).
- The solenoid valve, equipped with a flow sensor, was controllable, allowing selective entry from the inlet, either air or smoke, to the exposure chamber via specialized tubes.
- The sealed exposure chamber was made aerodynamically from a polymer material in a cylindrical shape, with a volume of 1.5 L that the removable specimens holder fitted in, and it was equipped with a negative pressure sensor. At the top of the exposure chamber, a humidifier was fitted in that sprays heated artificial saliva at (37 ± 1 °C), which was connected to a tank of saliva that integrated heater with a temperature sensor. The base of the exposure chamber features specifically fabricated pipes to channel the smoke or air to the negative pressure generator chamber for effective exhaust management (Fig. 3).
- The monitoring display shows the parameters of sensors in real time, as well as a control unit that controls all the electric components.
- The removable specimen holder was designed in a conical shape, with the specimens set around the base (Fig. 4). It is fitted securely at the bottom of the exposure chamber, with holes aligned to the opening of the tubes at the bottom of the chamber to allow even smoke or air distribution around the specimens. It holds a maximum of 28 specimens, ensuring that the polished surface of the specimen remains in direct exposure and is designed for uniform exposure, thus efficiently simulating experimental conditions. Three specimen holders (I: white, II: black, III: pink) were created to hold samples for each type of exposure (I: e-cigarette subgroup, II: c-cigarette subgroup, III: control subgroup). Each position in the specimen holder was numbered and had a group name (NS1, NS2, CD1, CD2, and so on).

**Fig. 5:**
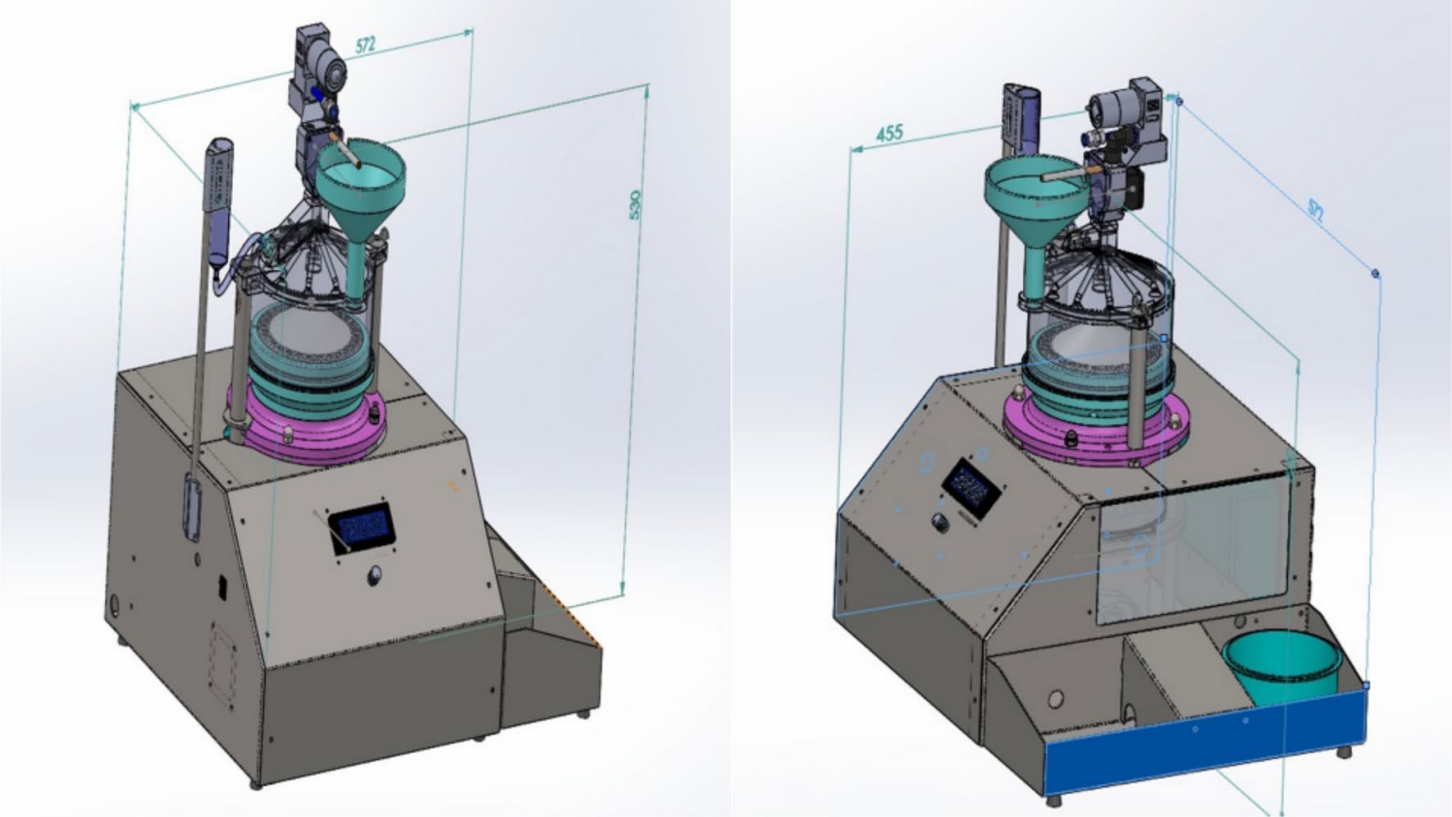
Machine design created by SolidWorks software. (This figure created by SolidWorks software version 2022 https://www.solidworks.com, displays the 3D model of the machine created using SolidWorks. The model shows the layout and dimensions of the machine, providing a detailed visualization of its structure. The design was simulated using CAD software to ensure that all parts fit together precisely and function as intended).

**Fig. 6:**
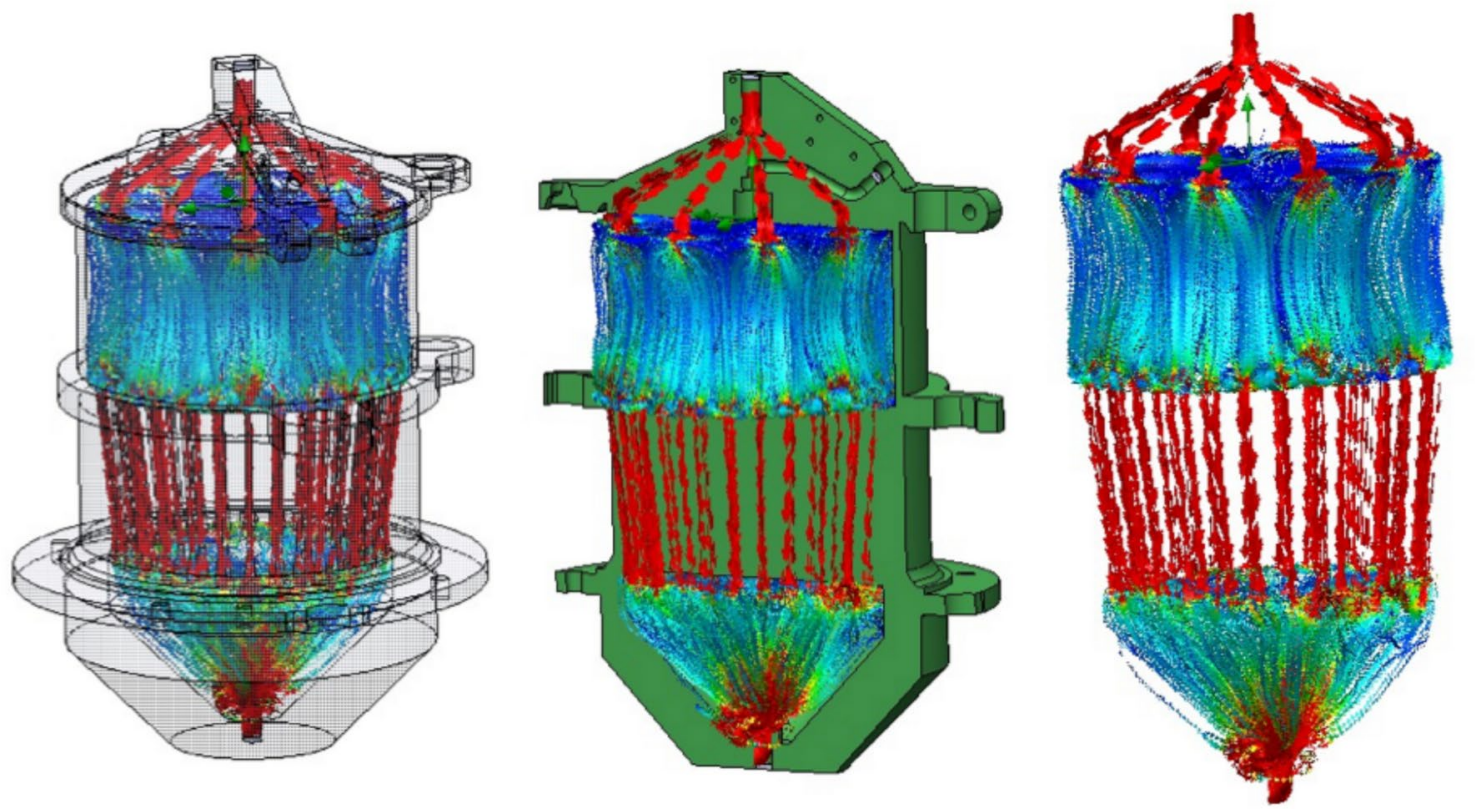
Aerodynamics simulations in the machine created by SolidWorks software. (This figure created by SolidWorks software version 2022 https://www.solidworks.com, shows the results of aerodynamics simulations performed on the machine’s components. These simulations were crucial for optimizing the smoke distribution within the exposure chamber. The results ensure that smoke is evenly distributed around all specimens for consistent testing conditions).

The vacuum pump creates negative pressure to draw smoke or air into the chamber, mimicking inhalation effects, and then the smoke/air enters the negative pressure chamber and is expelled outside by the vacuum pump. Such design elements ensure uniform smoke distribution in the chamber and consistency of the conditions during the exposure. The chamber and all of its parts were designed and tested in the aerodynamics simulations with computer-aided design (CAD) software SolidWorks (version 2022, Waltham, MA, USA) and then fabricated using 3D printing technology with filament material to ensure maximum precision and customization of parts (Figs. 5, 6).

**Fig. 7:**
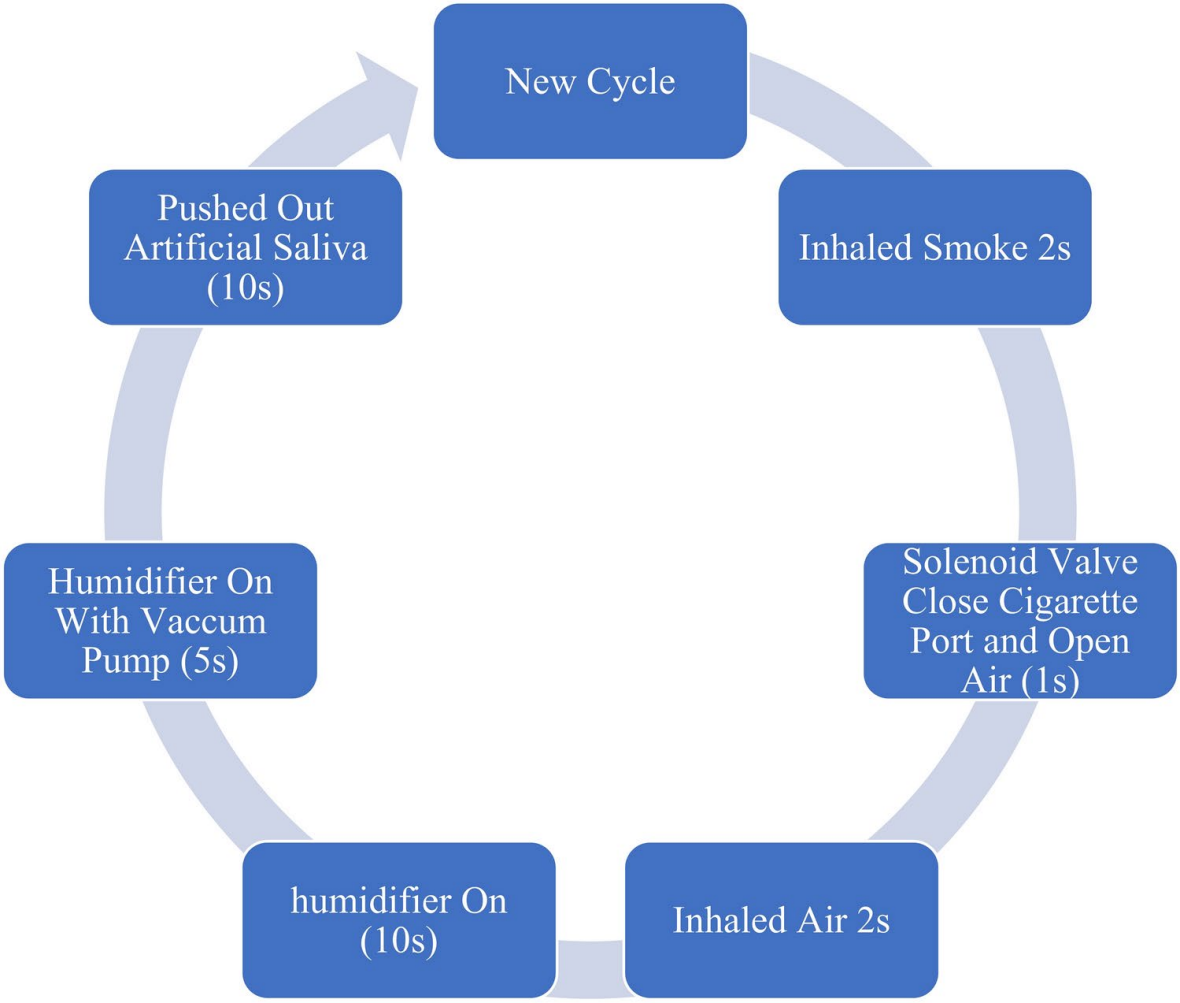
The smoking cycle. (This figure outlines the automated smoking cycle that the specimens undergo. The cycle consists of a 2-s inhalation phase followed by a 2-s air intake, between them 1-s for solenoid valve switching, and a 15-s period where temperature-regulated artificial saliva is sprayed into the chamber. The cycle repeats every 30 s, mimicking the habitual smoking patterns of a typical smoker).

The smoking cycle is automated and controlled by a pressure sensor, timer, solenoid valve, vacuum pump, and flow sensor. The cycle begins with a 2-s active inhalation of smoke at a steady flow rate of 30 cm^3^/s, creating a negative pressure of 20 mmHg inside the exposure chamber, as observed in typical smoking patterns from prior studies^14^, and aligns with ISO/TR 17219 standards. Afterward, normal air is drawn in for 2 s, followed by a 15-s spray of temperature-regulated artificial saliva from the humidifier. The vacuum pump then pushes out the humid air from the chamber for the same duration of inhalation before the next cycle, which repeats every 30 s^13^. Using a flow rate of artificial saliva that is similar to that of long-term smokers (0.3 ml/min)^29^, this cycle is made to mimic the smoking process over a long period of time (Fig. 7).

### Exposure to staining agent

After specimen preparation, measurements were taken for color at baseline (D1). Subsequently, the specimens underwent a staining process using the custom-made smoking simulation machine for 60 days. Then, after the staining procedure, the specimens were reevaluated for their color (D2). The exposure was designed to correspond to a heavy smoker’s life for six months^30,31^. Subgroups I were subjected to three cartridges of e-cigarettes per day for 60 days. Subgroups II were subjected to 60 cigarettes of c-cigarettes per day for 60 days. Subgroups III represented non-smokers, which constituted the control group. After exposure the specimens to the smoking, the specimens were under simulated oral hygiene practice by performing daily maintenance throughout the exposure time for 60 days. The daily brushing was performed in a total of three seconds for each specimen with an Oral-B PRO 1000 electric toothbrush equipped with a Cross Action brush head (Procter & Gamble Service GmbH, Schwalbach am Taunus, Germany). Brushing was performed with distilled water, and 0.4 g of Pronamel Daily Protection toothpaste (Sensodyne ProNamel, GlaxoSmithKline, Turkey) was applied on a pre-wetted brush head^32^. Subsequently, the specimens were washed with distilled water for one minute^14^.

After brushing, the specimens were kept in containers filled with artificial saliva within the incubator at a constant temperature of 37 ± 1 °C (the solution of artificial saliva was prepared in the Pharmaceutics Department at the Faculty of Pharmacy, Mansoura University, and contained 23% sodium biphosphate, 11.8% sodium chloride, 11.8% potassium chloride, and 29.5% urea with a pH of 6.8 ± 0.2). This protocol was designed to simulate the oral environment accurately and ensure durability and esthetic stability. The specimens were handled carefully to prevent scratches. The exposure to c-cigarette smoke stopped just 10 mm before filtering, and the device of e-cigarette was fully charged before use. The specimens were labeled by material type, exposure type, and number position. Artificial saliva was kept in the refrigerator and refreshed every 48 h to prevent microbial growth. The containers were covered to prevent evaporation of artificial saliva in the incubator^33^.

### Color change testing

The color parameters of all samples were measured at baseline and after exposure to the staining agent. These measurements were performed using a UV–Vis-NIR spectrophotometer (Cary 5000, Agilent Technologies, Santa Clara, CA, USA). Color differences (ΔE_ab_*) were calculated between (D1–D2), using the CIE-L*a*b* color space system:

$$\Delta {\text{E}}_{*} = \sqrt {\left( {\Delta L_{*} } \right)^{2} + \left( {\Delta a_{*} } \right)^{2} + \left( {\Delta b_{*} } \right)^{2} }$$

Values were established within the mathematical coordinates of the international color space CIE-Lab (Commission International de l’EclairageL*a*b*), which defines a color space by three independent variables: L*, a*, and b*. The L* coordinate depicts the luminosity or lightness of the color, ranging from black (L* = 0) to white (L* = 100). The a* and b* coordinates describe the chromaticity of the color, with the a* axis ranging from green to red and the b* axis from blue to yellow. This color space is represented on a sphere in which the Y-axis is the L* coordinate, the X-axis is the b* coordinate, and the Z-axis is the a* coordinate. This system enables the exact and quantitative comparison of colors^24^.

### Statistical analysis

The data were analyzed using the software SPSS (version 25, SPSS Inc., Chicago, IL, USA).

## Results

A Shapiro–Wilk test was used to diagnose the normality of the data distribution of all variables. The data were parametric and met the normal distribution. Data were expressed as mean ± SD. Color change data between and within groups (NS and CD) and subgroups (I, II, III) were compared by 2-way ANOVA followed by the Bonferroni test for multiple comparisons. The graphic presentation of data was done using clustered bar charts. The *p* < 0.05 was considered to be significant.

The two-way ANOVA for color change presented in Table 2 yields a significant difference for more than one factor. First, there is a statistically significant difference in color change between the groups, F(1,30) = 345.83, *p* < 0.001, showing that the two groups tested are statistically different in their color changes. Overall, very significant subgroup differences existed in the color change as F(2, 30) = 6452.97, *p* < 0.001, indicating that differences contributed by subgroups are very important. Finally, there was a significant interaction effect of group and subgroup: F(2,30) = 43.93, *p* < 0.001, which means that the effect of one factor depends on the level of the other, or in other words, the response in color change varies not only between groups but also across subgroups within those groups.

**Table 2:** Summary of two-way ANOVA of color change.

Source	Type III sum of squares	df	Mean square	F	Sig
Group	3.99	1.00	3.99	345.83	< 0.001*
Subgroup	148.78	2.00	74.39	6452.97	< 0.001*
group * subgroup	1.01	2.00	0.51	43.93	< 0.001*
Error	0.35	30.00	0.01		

*p is significant at 5 levels of significance.

### Effect of the group on color change

**Fig. 8:**
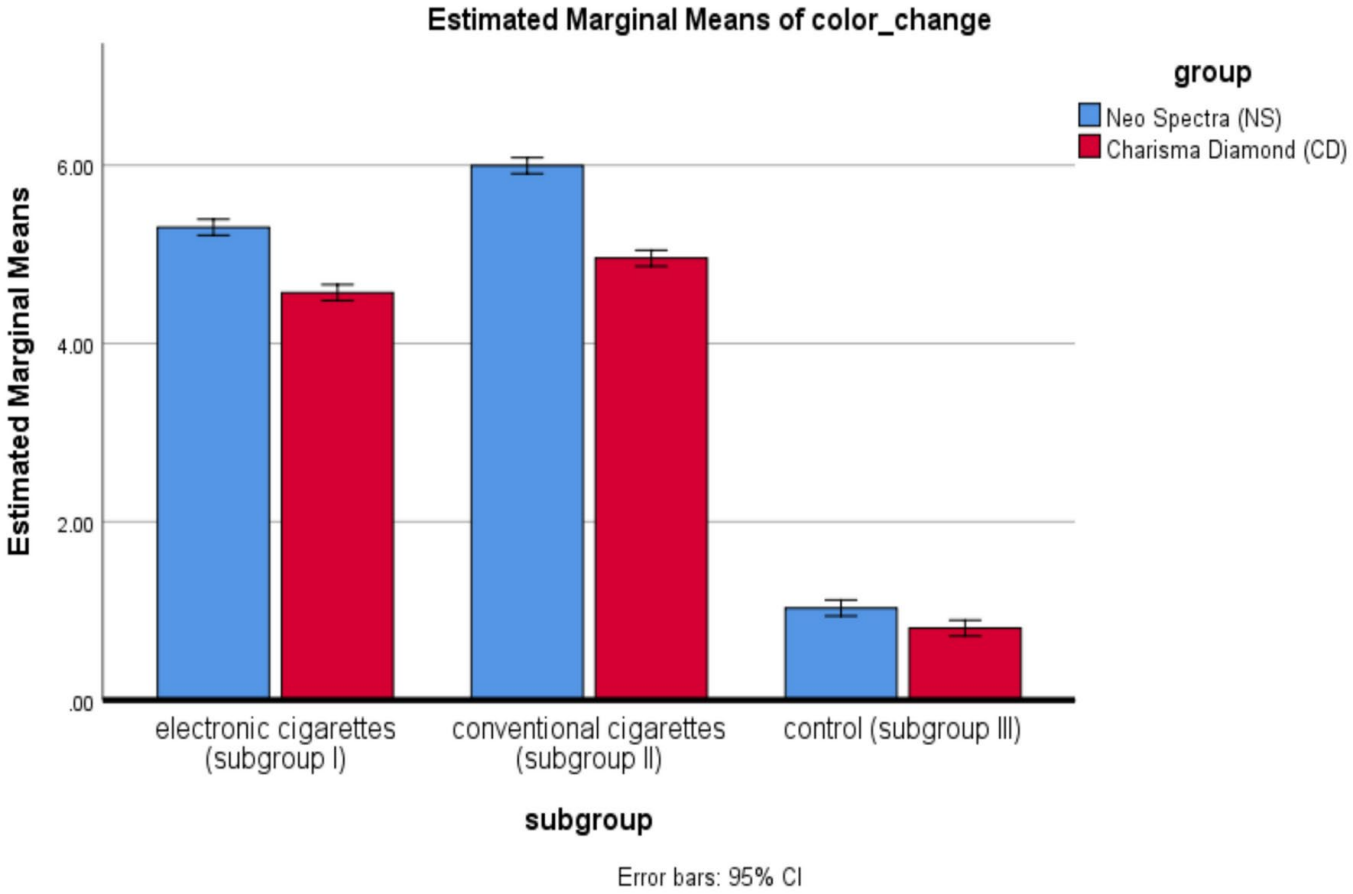
Comparison of color change between groups for different subgroups. (This figure compares the color change (ΔEab) between the two main groups, Neo Spectra ST-HV (NS) and Charisma Diamond One (CD), across the three subgroups (electronic cigarette, conventional cigarette, and control). It visually represents the degree of color change observed for each material under different smoking conditions, highlighting significant differences between the groups).

**Fig. 9:**
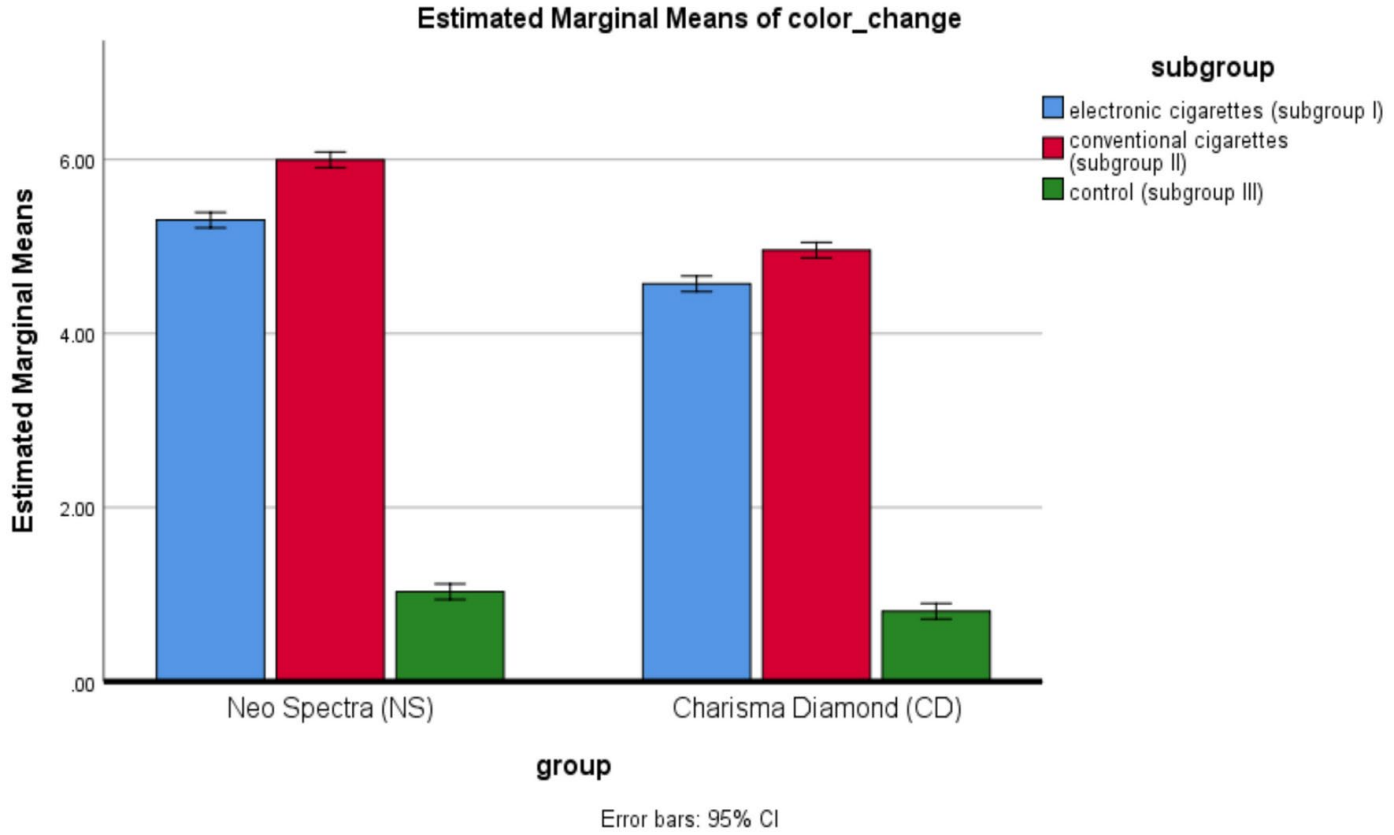
Multiple comparison of color change between each subgroups for different groups. (This figure presents a more detailed comparison of color change between the subgroups for both NS and CD groups. This figure shows how the color change varied across the different exposure conditions (e-cigarette, conventional cigarette, and control) for each material. The data highlights which exposure condition had the most significant impact on the color stability of the restorative materials).

Table 3 and Fig. 8 show the color change comparison between the two groups, NS and CD, for each subgroup. The results have shown that in all the subgroups tested, the color change is highly significant. In Subgroup I, NS recorded a higher color change than CD at *p* < 0.001. Similarly, in Subgroup II, NS recorded more color change than CD at *p* < 0.001. In Subgroup III, NS also revealed a significantly higher color change than did CD, *p* = 0.001. These results show that NS produced a higher color change than that of CD within all subgroups, as depicted in Fig. 9.

**Table 3:** Comparison of color change between groups and subgroups.

Materials	Electronic cigarette Subgroup I		Conventional cigarette Subgroup II		Control Subgroup III		Two-way ANOVA (*p* value)	Electronic cigarette -conventional cigarette	Electronic cigarette-control	Conventional cigarette-control
	X	*SD*	*X*	*SD*	*X*	*SD*				
Neo Spectra ST_HV Group (NS)	5.30a	0.14	5.99b	0.13	1.03c	0.07	< 0.001*	< 0.001*	< 0.001*	< 0.001*
Charisma Diamond One Group (CD)	4.57a	0.10	4.96b	0.10	0.81c	0.09	< 0.001*	< 0.001*	< 0.001*	< 0.001*
Independent samples t-test (p-value)	< 0.001*		< 0.001*		0.001*					

X; ΔE mean, SD; standard deviation; *p is significant at 5% level. Different letters in the same raw showed a significant difference between each 2 subgroups (Bonferroni test, *p* < .05). Similar letters in the same raw showed non-significant difference between each 2 subgroups (Bonferroni test, *p* > .05).

### Effect of subgroup on color change

The comparison of color change between subgroups for each group is shown in Table 3 and Fig. 8, which presents that there was a statistically significant difference in the magnitude of color change for both materials. Maximum color change was observed in Subgroup II, followed by Subgroup I, while minimum in Subgroup III. Several post-hoc multiple comparisons, represented by Table 3 and Fig. 9 below, indicated all the subgroup pairs were statistically significant to further affirm this trend observed in color change variation from subgroup severity: In detail, these results represent an absolute tendency towards changeability; among those changes, subgroup II presents the largest one.

## Discussion

To address the gaps with prior investigations, this research developed a custom-made smoking simulation machine, specifically for dental material testing under controlled smoking conditions, that addresses critical limitations of the existing devices. The salient features of this machine are the artificial saliva with temperature control, programmable smoking cycles, and controlled aerodynamics that are truly unique and provide a realistic and repeatable test environment. The precision in the condition of exposure, which this machine provides, ensures that even long-term experiments could be conducted to simulate actual smoking effects on dental restorative materials. These automated smoking cycles are models of natural smoking patterns, and the uniform distribution of smoke or air ensures that all the specimens have identical exposure, enhancing the reliability of the results^19^.

Color change in dental restorations is crucial for esthetics and durability. Intrinsic factors like resin matrix type, filler particle size, and photoinitiator system affect color change. Incomplete polymerization also affects color change. Extrinsic factors like poor oral hygiene, dietary habits, smoking, and mouth rinses also pose threats to color change. These factors influence the overall quality and durability of dental restorations^24,34^. This study focuses on nanohybrid RBCs, which are widely used in dental clinics for restorations in both anterior and posterior teeth, demonstrating superior physicochemical and mechanical properties^35^. Single-shade RBCs feature a universal shade that matches all 16 VITA Classic shade tabs. On the other hand, multi-shade composites offer a limited range of shades designed to match specific shade tab sets^36^. The A2 shade is widely used and considered a standardized shade for composite samples, allowing better color evaluation and minimizing influences on variation^37^.

E-cigarettes were used in this investigation due to their market dominance, standardized nicotine content, consistent vapor production, and chemical composition, making them representative of real-world usage^38^. Also, c-cigarettes were included in this study due to their global popularity, standardized nicotine and tar content, and strong relevance in studying the health and material impacts of conventional smoking^39^. While artificial saliva was utilized to standardize stimulation of oral conditions, it enabled extensive research on RBCs’ properties and the development of durable, esthetic dental restorations^40^. The inclusion of a control group in this study provides a baseline for comparison, allowing researchers to attribute observed outcomes to the intervention, minimizing potential biases, and strengthening internal validity, thereby establishing causal inferences^41^. Serially sealed, numbered, opaque envelopes were utilized to ensure proper randomization, maintain allocation concealment, and prevent selection bias^42^.

Teflon molds were utilized due to their non-sticking, chemical inertness, thermal stability, and resilience, ensuring easy sample removal, contamination prevention, and precise specimen shaping^43^. Also, Teflon molds are partially translucent; light penetrates deeper and may overestimate the depth of cure (DOC) because more light may activate the resin composite than normally would occur in an opaque mold^44^. The study utilized a disk-shaped specimen with a diameter (of 10 mm), which varies based on the instrument’s reading aperture, with a thickness (2 mm) that is less than 3 mm for color change detection^45^. In the present study, transparent celluloid strips in RBCs specimen preparation were used to create a smooth surface, prevent an oxygen-inhibited layer from forming during polymerization, and standardize surface characteristics between samples to maintain consistent experimental conditions^23^.

In the current examination, a strip and glass slab with a 500-g weight were applied for 20 s to ensure standard pressure, avoid contamination, and distribute stress, achieving uniformity in smooth specimens^22^. All the specimens in this investigation were polymerized uniformly (as recommended by each manufacturer). While curing the composites against a transparent strip without subsequent finishing, a resin-rich surface is obtained, and this surface is more prone to discoloration. Finishing results in a filler-rich surface with enhanced hardness and reduced susceptibility to chemical breakdown^46^.

Allowing 24 h for post-polymerization increases the degree of polymerization, reducing the elution of unreacted monomers and improving biocompatibility, mechanical strength, and esthetics^24^. The incomplete polymerization of RBCs can lead to increased water absorption and solubility, resulting in early color instability^47^. In addition, the delayed finishing and polishing offer better color change and mechanical properties, making it ideal for durable and consistent dental restorations with good appearance^25^. All specimens were finished and polished by the same operator for standardization^34^. The research utilized a one-step finishing and polishing system for RBCs disc surfaces, resulting in smooth, durable, and esthetically pleasing surfaces that are easy to use, resistant to discoloration and plaque accumulation, and enhance hygiene^48^.

The specimens in the present study were stored at 37 °C for 60 days, which simulated exposure to smoke for a six-month clinical period of a heavy smoker’s life. Each day of exposure was designed to correspond to three days of heavy smoking, as a heavy smoker would consume one cartridge per day of e-cigarettes (5% nicotine cartridge) or twenty cigarettes of c-cigarettes^30,31^. The control subgroup was incubated in artificial saliva, while other subgroups were exposed to smoking e-cigarettes or c–c-cigarettes in addition to being incubated in artificial saliva at 37 °C. This study addressed a gap in prior research by incorporating tooth brushing, a critical factor in reducing staining^45^, and using a low abrasive relative dentin abrasivity toothpaste, which was employed in this study due to its common use^49^. This work guaranteed the standardization of methods, as all the steps were made by the same operator. Color parameters were considered in two-time frames: D1 (base measurements) and D2 (after exposure). This method allowed for a controlled environment to measure the effects of smoking on RBCs.

The study utilized instrumental spectrophotometry to assess color changes in RBCs, potentially reducing subjective errors in color evaluation. Instrumental methods, such as colorimetry and spectrophotometry, are reliable in dental material research. Absolute measurements are quantified as ΔE_ab_, and if a material is color stable, no variation is observed (ΔE_ab_* = 0)^50^. The color change of dental composites was evaluated on a ΔE_ab_ evaluation scale quantifying perceptibility and acceptability based on the following criteria: A ΔE_ab_ value of 1 unit or less represents a non-visible difference, indicating that the change is not visible to an average observer. A value in the range of 1–2 units is considered visually perceivable, noticeable by a trained examiner, but may not be evident to an untrained observer. A ΔE_ab_ of exactly 3.3 units is defined as clinically acceptable, meaning that it is a visible change that is still acceptable for clinical use. It, therefore, becomes important to differentiate between clinically insignificant and clinically significant color changes, hence providing a clear benchmark in esthetic integrity evaluation for materials under study conditions^18,24,51^. The study found that both types of smoking used in the current study caused a significant change in the color of tested restorative materials, which was clinically unacceptable due to higher ΔEab values than the maximum acceptable limit (more than 3.3), while control subgroups showed clinically acceptable color changes (less than 3.3).

Results obtained in this study showed that c- cigarettes smoking causes a higher grade of discoloration of restorations compared to the use of e-cigarettes, which had an unacceptable discoloration. These results were in agreement with Alnasser et al.^16^, Paolone et al.^6^, and Abdulla et al.^5^, supporting the fact that smoking remains one of the major causative agents in dental discoloration. While these results disagree with the findings reported by Vohra et al*.*^13^ that claim e-cigarettes cause similar discoloration to c-cigarettes, this disagreement may stem from the use of different smoking materials in their study or the machine used in exposure. Several reasons could also be considered for these differences, including the measurement techniques used, the type of materials used in each kind of cigarette, and the usage patterns during experiments or the machine used.

Tar in c-cigarette smoke is most injurious due to its dark pigmentation and very sticky nature; hence, it is considered a major cause of discoloration. In addition to the aforementioned, the combustion of tobacco also produces other substances responsible for stains, such as sugars and additives like cocoa. Thermal effects of burning tobacco lead to changes in the color of restorations. While e-cigarette smoking may allow for some discoloration, it is way less intense compared to cigarette smoking. This underlies some of the distinctive physical and chemical properties that underlie the staining propensity of cigarette smoke^17^.

It is shown in this study that NS RBCs are more susceptible to staining and have higher color changes compared to CD RBCs. These results disagree with Maghaireh et al.^52^, who reported NS RBCs showed more color stability than CD RBCs; this disagreement might be due to different staining agent materials than the current study or different finishing and polishing systems used in the present study. These higher staining susceptibilities are mostly due to the use of different resin matrix compositions and types of filler particles in the two RBCs. Significantly, it is the resin component that usually plays the most important role in determining susceptibility to staining^53^. The increased stability in CD RBCs can be accredited to the large molecular size of TCD-urethane and the absence of diluting agents. The larger molecular size and complete avoidance of diluting agents allow for increased chemical stability because there is less chemical reactivity or degradation^54^.

Enhancing this stability further are the formulations combining UDMA with TCD, TCD-DI-HEA, which reach higher degrees of polymerization and maintain lower residual double bond concentrations than the Bis-GMA-based composites, a prevalent component of NS RBCs materials. Furthermore, the UDMA/TCD-DI-HEA combination in CD RBCs provides low viscosity and low water absorption, which is instrumental in the improvement of color stability and mechanical integrity. These are desirable properties for dental materials that need to withstand the hostile oral environment^55^. On the other hand, the hydrophilic nature of the Bis-GMA matrix present in NS RBCs results in higher water sorption, which ultimately imparts lower stain resistance when compared to other methacrylate monomers. At the same time, the addition of TEGDMA to a resin matrix at an incremental rate from 0 to 1% increases the water uptake of Bis-GMA-based resins from 3 to 6%^47,56^. The study emphatically established that NS RBCs material containing both Bis-GMA and TEGDMA exhibits a higher color change than CD RBCs.

The RBCs examined in this study, NS and CD, were both nanohybrid RBCs utilizing the same photoinitiator system. The color of dental RBCs is highly influenced by the fillers of the RBCs^57^. Notably, the filler content for the CD RBCs was marginally higher. Filler content is considered one of the most significant factors that influence the stain absorbency of resin composites. Several studies have pointed out that increasing the filler content normally reduces stain absorbency, most likely due to the decrease in the resin matrix surface area^47,58^.

From the results of the research, the small color change variability among the samples with a small standard deviation would indicate that the materials were equally exposed to cigarette smoke and that it caused equal staining effects, although each sample was allocated in a different position within the specimen holder. The fact that exposure was similarly maintained in the course of processing could be explained by controlled aerodynamics in the distribution of smoke particles during the exposure process, which showed that the machine of exposure was accurate.

## Limitations

The study has limitations in using limited types of smoking sources, and RBCs materials in controlled laboratory settings do not fully simulate the complexity of oral environments. Besides, while the focus of this study is on color changes, mechanical strength, and surface roughness, which are equally important properties, they are not discussed. While machine smoking provides repeatable data for laboratory use, its inability to mimic real human smoking behaviors calls for updated testing protocols that account for enzymatic activity and other in vivo conditions affecting material degradation in smokers.

## Conclusions

Within the limitation of this research, the following conclusions could be drawn:

- This custom-made smoking simulation machine provides a standardized and controlled method for evaluating the effects of smoke on dental restorative materials, offering advanced features such as temperature-regulated artificial saliva and precise aerodynamics to simulate real-world smoking conditions.
- Conventional cigarette smoking causes more discoloration than electronic cigarette smoking. On the other hand, the response to smoke exposure varied by material type; specifically, NS RBCs exhibited more discoloration than CD RBCs under all smoking conditions.

## Recommendations

The machine can be used to test restorative materials like ceramics, glass ionomers, and dental prosthesis materials with programmable features while maintaining consistent aerodynamics. Its versatility could easily allow comparative studies on the effects of tobacco products on the long performance and durability of dental materials. It could also be further improved by the inclusion of biological factors involved in material degradation in vivo, such as enzymatic activity, microbial colonization, or changes in saliva pH. The inclusion of a model of the human jaw within the machine could also provide more realistic conditions. The smoking cycle in this machine may not be able to capture all the variations in smoking behaviors among individuals. The test methods, however, require updating with the types of cigarettes evolving and ways of smoking, and more research needs to be done on smoking cycles. Future improvements could include variable flow rates or custom smoking profiles for different scenarios. This would provide a deeper understanding of the influence of various smoking habits on dental materials and would also make the machine more capable by providing clinically relevant data that guides material development and innovation.

The machine should be calibrated to simulate, through Boyle’s Law, the pressure–volume relationship in the human oral cavity upon inhalation in order to produce more realistic smoking simulations. This design should allow progressive chamber pressure variation, ensuring that volume increases with pressure drop. This dynamic simulation should involve both steady-state and dynamic pressure changes to enhance material testing accuracy. Advanced in vitro models, with more research, require thorough knowledge about the pressure–volume relationship of the oral cavity and thus provide better means to test tobacco products. Finally, although the in vitro model is helpful, it cannot actually simulate the oral environment. The long-term performance and durability of dental materials exposed to smoking will be determined through in vivo studies investigating the changes in material degradation over time influenced by smoking and natural oral processes that may affect color stability, surface roughness, and other properties. Both in vitro and in vivo approaches would, when combined, provide more clinically relevant data.

## Data Availability

The data that support the findings of this study are available from the corresponding author, upon reasonable request.
